# Salovum Egg Yolk Containing Antisecretory Factor As an Adjunct Therapy in Severe Cholera in Adult Males: A Pilot Study

**DOI:** 10.3329/jhpn.v29i4.8443

**Published:** 2011-08

**Authors:** Nur H. Alam, Hasan Ashraf, Maryam Olesen, Mohammed A. Salam, Niklaus Gyr, Remy Meier

**Affiliations:** ^1^icddr,b, GPO Box 128, Dhaka 1000, Bangladesh; ^2^Novartis Consumer Health, Bern, Switzerland; ^3^University Hospital Basel, Basel, Switzerland; ^4^Gatroenterology Department, University Hospital, Liestal, Switzerland

**Keywords:** Antisecretory agents, Cholera, Salovum egg yolk powder, Bangladesh

## Abstract

Cholera involves stimulation of intestinal secretory process in response to cholera toxin leading to profuse watery diarrhoea that might cause death due to dehydration unless timely rehydration therapy is initiated. Efforts to identify and test potential antisecretory agents are ongoing. Antisecretory factor (AF) is a naturally-occurring protein produced in the human secretory organs, including the intestine, with antisectory properties demonstrated in animal and human models of secretory diarrhoea. Salovum egg yolk powder contains proteins with antisecretory properties in a much higher (500 times) concentration than that of normal hen eggs. This is achieved by feeding hens with specially-processed cereals, capable of inducing proteins with antisecretory properties in the yolk. The aim of the study was to examine the effect of Salovum egg yolk powder containing AF in the treatment of adult cholera patients. In an open, randomized controlled trial (pilot study), 40 adult male patients with severe cholera were studied: 20 received standard treatment (oral rehydration solution, antibiotic, and usual hospital diet) plus Salovum egg yolk powder (study group) and 20 received standard treatment alone (control group). All the patients received tablet doxycycline (300 mg) once immediately after randomization. Written informed consent was obtained from each subject before enrollment. The main outcome measures were stool weight and duration of diarrhoea. The demographic and baseline clinical characteristics of the study patients were comparable between the groups. No significant differences were found in the mean stool weight, g/kg of body-weight during the first 24 hours [study vs control group, mean±standard deviation (SD), 218±119 vs 195±136], second 24 hours (mean±SD, 23±39 vs 22±34), and cumulative up to 72 hours (mean±SD, 245±152 vs 218±169). The duration (hours) of diarrhoea after admission in the hospital was also similar in both the groups (mean±SD, 33±14 vs 32±10). No adverse effect was observed. Salovum egg powder containing AF as an adjunct therapy in the treatment of severe cholera could not demonstrate any beneficial effect. Further studies with higher doses of Salovum egg yolk powder might be considered in future to establish its antisecretory effect.

## INTRODUCTION

*Vibrio cholerae* is the enteric bacterial pathogen that causes cholera. The disease can often be very severe characterized by frequent passage of voluminous watery stools and vomiting leading to severe dehydration and, if not efficiently treated, might result in death; the rates can be as high as 50-80% (1). Prevention of dehydration, rehydration using appropriate oral or intravenous fluids, and the use of an effective antimicrobial agent, along with continued feeding, are important in the case-management strategy of cholera (2,3). Since cholera involves stimulation of secretory process in response to cholera toxin produced by *V. cholerae*, efforts to identify and test potential antisecretory agents to reduce the severity of diarrhoea are ongoing. Most of these agents assessed so far have no or minimal effect (4-15). Until now, none of the antisecretory agents has been recommended for the clinical management of cholera patients.

As possible antisecretory agents, some endogenous factors have recently drawn the attention of researchers. Antisecretory factor (AF), a naturally-occurring protein, is produced in the brain and in the secretory organs, such as gallbladder, lungs, kidneys, and the intestine, in response to infection (16-20). AF is also secreted in blood, bile, and breastmilk in response to intestinal enterotoxin challenge (21), and the content in sows’ milk is probably crucial for protection against neonatal diarrhoea in suckling piglets (22). It has also been found that carbohydrates and amino acids of specially-processed cereals are also able to induce secretion of AF or AF-like proteins (23,24), which might be useful for therapeutic applications. Preclinical animal studies have shown that AF can have both prophylactic and therapeutic applications in the treatment of various types of diarrhoea (25-29). Results of human studies also demonstrated that AF possesses strong anti-inflammatory properties (30-32).

The Salovum egg yolk powder contains protein with antisecretory properties in a much higher (500 times) concentration than found in normal hen eggs. This is achieved by feeding hens with specially-processed cereals, capable of inducing production of protein with antisecretory properties in the yolk, from which an egg powder is produced (33,34). In view of its apparent antisecretory and anti-inflammatory effects, AF could be therapeutically useful in diarrhoeal diseases of various aetiologies. Therefore, we evaluated the effect of salovum egg yolk powder containing AF in the management of adult male cholera patients.

## MATERIALS AND METHODS

This open, controlled (pilot study) clinical trial was conducted among 40 adult patients to evaluate the effect of AF-rich Salovum egg yolk powder as an adjunct therapy in the management of severe cho-lera. The study patients were selected from among those attending the Dhaka Hospital of icddr,b from June to December 2005. The Ethics Review Committee of icddr,b approved the protocol. Adult male patients who attended the Dhaka Hospital for treatment were screened for eligibility. All patients aged 18-55 years with a history of watery diarrhoea of <24 hours and signs of severe dehydration and initial stool dark-field microscopy positive for *V. cholerae* were eligible for participation in the study. Patients with a history of chronic diarrhoea, dysentery, receiving antimicrobial or antidiarrhoeal drugs within one week before admission, renal or hepatic dysfunction, known allergies to eggs, and refusal of written informed consent were excluded from the study. Upon initial screening, patients were brought to the research ward of the hospital, weighed, and placed on a cholera cot. A standard medical history, thorough physical examinations, including assessment of dehydration using the modified guidelines of the World Health Organization (WHO) (35), and vital signs were recorded in pre-designed forms. Before randomization in the study, all the patients were rehydrated with intravenous (IV) fluids containing polyelectrolytes (Na 133, Cl 98, K 13, and acetate equivalent to 48 mmol/L of bicarbonate) at a rate of 100 mL/kg of body-weight over 4-6 hours, in addition to replacement of ongoing losses in stool and vomit.

**Fig. 1.**
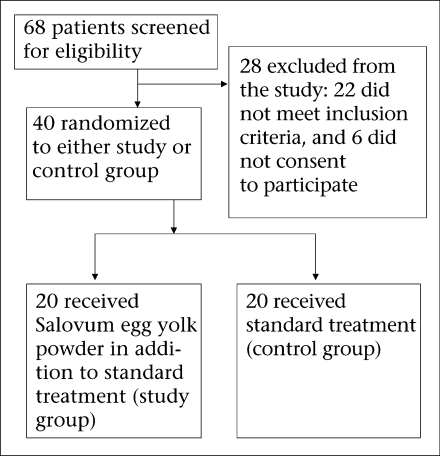
Trial profile

The enrollment of patients in the study was done every day from 6 am to 2 pm when most severely-dehydrated patients attend the hospital. In total, 68 adult male patients with suspected cholera were screened at the hospital, of whom 40 were randomized to treatment (trial profile in Fig. 1). The main reasons for the exclusion of 28 patients were: 22 being *V. cholerae* negative by dark-field microscopy and six refused to provide consent to participate in the study.

### Randomization

Eligible patients who fully rehydrated within 4-6 hours were randomized in equal numbers to receive: (a) AF (2 sachets of Salovum egg yolk powder—2 g each, i.e. total 4 g, were dissolved in 100 mL of oral rehydration salt solution (ORS) and fed orally every two hours during the first 24 hours and then four hourly until the resolution of diarrhoea but up to a maximum of 72 hours), in addition to standard treatment (ORS, antibiotic, and usual hospital diet) or (b) the standard treatment (ORS, antibiotic, and usual hospital diet) alone. The dose decided was partly arbitrary and was based on a previous study among patients with inflammatory bowl disease; the dose used 4 g four hourly for 14 days. Since cholera is an acute severe disease, we decided frequent dosing (2 hourly) at least for 24 hours. A trained staff of icddr,b, not involved in the study, prepared the randomization list, and the name of intervention was indicated on a paper, kept inside the sealed envelopes. The sealed envelopes were kept with the pharmacists of the Dhaka Hospital as they were not involved in the study. After receiving the name, the study serial number, and the hospital number of the patient to be randomized, the pharmacist opened the sequentially-numbered sealed envelopes, starting with the lowest number and moving towards higher numbers and supplied the sachet of Salovum egg yolk powder or informed about control (standard treatment alone).

### Case management

Immediately after randomization (following IV rehydration), maintenance oral rehydration therapy was started using the WHO-recommended ORS (Na 75, glucose 75, Cl 65, K 20, and citrate 10 mmol/L and osmolarity 245 mosmol/L). During maintenance, patients consumed ORS according to need, with minimum volume equal to continued loss of watery/loose stool and vomit. All the patients received capsule doxycycline (300 mg) as a single dose within 20 minutes of randomization into the study. A standard diet was provided according to the hospital practice (breakfast at 6:30 am, lunch at 12 noon, and supper at 6 pm). Plain water was allowed as desired by the patients preferably after food. IV fluid therapy was restarted in some patients where signs of severe dehydration reappeared despite appropriate ORS therapy or where excessive vomiting prevented successful oral therapy. Stool was collected in a bucket placed underneath the cholera cot. All intakes (IV fluid, ORS, and water) and outputs (stool, urine, and vomit) were measured and recorded every six hours during the study until discharge. Weight of stool was measured with an electronic scale with a precision of 1 g (Sartorius, Germany). Urine was separated in a urine collector and measured with a calibrated cylinder. Weight of vomit was measured by collection in a pre-weighed bowl by subtracting the weight of the bowl. The volume of ORS and plain water consumed by the patients was measured with a calibrated cylinder. Body-weight was measured on admission (before initiation of IV therapy), at randomization (after IV rehydration), and every six hours until discharged, using the same electronic scale.

### Laboratory investigations

Fresh stool samples were examined for *V. cholerae* by dark-field microscopy during the initial 4-6-hour IV rehydration period. Stool samples were cultured for the isolation and identification of *V. cholerae, Salmonella, and Shigella* using the standard techniques at randomization. Peripheral venous blood samples were tested for haematocrit, total and differential white blood cells, blood urea nitrogen, creatinine, serum sodium, potassium, chloride, and bicarbonate at randomization and after 24 hours.

All the patients were observed closely until discharge. Resolution of diarrhoea was defined if patients did not pass any stool for at least 12 hours or if they passed two consecutive normal (formed) stools. Clinical success was defined as cessation of diarrhoea within 72 hours from the start of study medication, and those with continued watery stool for more than 72 hours were considered clinical failure. Oral therapy failure was defined as reappearance of signs and/or symptoms of dehydration, requiring unscheduled IV fluid therapy. The duration (hours) of diarrhoea was calculated from the time of randomization to the last watery stool.

### Analysis of data

All statistical analyses were performed using the SPSS PC+ software (version 10). All the statistical tests were two-tailed, performed at 5% level of significance. The continuous variables were compared using the Student's *t*-test, and the categorical variables were compared with the χ^2^ test or Fisher's exact test as appropriate. Kaplan-Meier survival curves were constructed for the duration of diarrhoea and was compared by log rank test.

## RESULTS

**Fig. 2.**
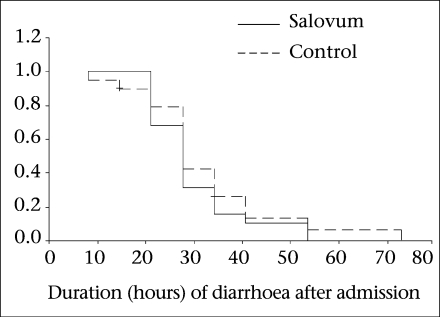
Kaplan-Meier survival curve for duration of diarrhoea

The demographic and baseline clinical characteristics with regard to the patients’ age, body-weight, duration of diarrhoea before admission, duration of vomiting, stool (g)/kg of body-weight per hour during the IV rehydration period were comparable between the groups (Table 1). There were no significant differences between the two treatments for the major outcome variables: stool output, ORS intake, and need for unscheduled IV fluids (Table 2). The mean duration of diarrhoea was also similar in both the groups. Kaplan-Meier survival curves (Fig. 2) also showed a similar pattern in both the groups (p=0.83, log rank test). Serum electrolytes, blood urea nitrogen, and creatinine were within normal limits and were similar in both the groups (data not shown). No adverse experience, such as abdominal pain, any allergic skin manifestation, etc., was noted during the study.

**Table 1.** Demographic and clinical characteristics of study patients on admission

Variable	Salovum egg (mean±SD) (n=20)	Control (mean±SD) (n=20)	p value
Age (years)	23.65±4.80	24.90±6.28	0.25
Body-weight (kg) on admission	45.25±5.10	43.86±4.62	0.96
Duration (hours) of diarrhoea before admission	10±5	10±5	0.88
Number of stools before admission	14±9	15±8	0.57
Duration (hours) of vomiting before admission	7±4	8±5	0.26
Number of vomits before admission	8±7	10±8	0.42
Number of stools during the observation period	15±9	10±7	0.42
Received IV fluids (mL/kg of body-weight) during the IV rehydration period	192±28	181±37	0.27
Stool rate during the IV rehydration period (g/kg of body-weight/hour)	13.44±7.68	10.41 ± 7.55	0.60

IV=Intravenous;

SD=Standard deviation

**Table 2.** Comparison of outcome variables of study patients

Variable	Salovum egg (mean±SD) (n=20)	Control (mean±SD) (n=20)	p value
Stool weight (g) on day 1/kg of body-weight	218±119	195±195	0.57
Stool weight (g) on day 2/kg of body-weight	23±39	22±34	0.87
Stool weight (g) cumulative/kg of body-weight up to 72 hours	245±152	218±169	0.60
ORS (mL) intake on day 1/kg of body-weight	279±107	245±108	0.32
ORS (mL) intake on day 2/kg of body-weight	42±54	46±59	0.82
ORS (mL) intake cumulative up to 72 hours/kg	325±143	295±169	0.55
Duration (hours) of diarrhoea	33±14	32±10	0.64
Number of patients received unscheduled IV fluid*	5 (25)	4 (20)	0.70

* Values are number (%);

IV=Intravenous;

ORS=Oral rehydration salt solution;

SD=Standard deviation

## DISCUSSION

The results of the study demonstrated that Salovum egg yolk powder rich in AF used as an adjunct therapy as standard treatment of severe cholera failed to show any additional beneficial effect. It neither reduced the severity of illness by reducing stool output nor reduced the duration of diarrhoea. Although results of studies in animal models showed its antisecretory effect, its efficacy in patients with secretory diarrhoea due to microbial pathogens was not previously proven (25-29). The reasons why the Salovum egg yolk powder containing AF did not work cannot be explained from this study. We can assume the possible causes as: (a) degradation of the protein with antisecretory properties while passing through the stomach to the small intestine, the main site of its action; (b) failure to work for the nature of the disease process in severe cholera where the intestinal transit time is very short and the material might have washed out before it could act; and (c) the dose of Salovum egg yolk powder used might not be adequate. Therefore, further clinical studies with larger doses might be planned in future. Recently, one study has shown the beneficial effect of Salovum egg yolk powder in terms of reducing the stool frequency and early recovery of children suffering from acute (duration of diarrhoea <7 days) and prolonged (duration of diarrhoea >7 days) non-cholera watery diarrhoea (36). However, this study is not comparable with our study, especially in relation to the study subjects, aetiology, and pathophysilogy. It may be mentioned that the patients in the Salovum group had a greater frequency of stools and a higher stool volume (Table 1). during the observation period compared to the control group, although these trends were not significant. After intervention, the patients in the Salovum group had a higher stool volume on day 1, 2, and cumulative up to 72 hours and greater ORS intake on day 1 and cumulative up to 72 hours. They also had slightly longer duration of diarrhoea and an increased number of patients receiving unscheduled IV fluid compared to the control group but these trends were also not significant (Table 2). If the Salovum group did have patients who had a more severe state of cholera at baseline than the control group, this might conceal any beneficial effects of Salovum egg yolk powder.

### Conclusions

We conclude that the use of Salovum egg yolk powder containing AF apparently did not provide any additional benefit in the treatment of severe cholera in adults. Although this was found to be safe for short-term evaluation, we do not have any information on long-term adverse effects. Further studies with a larger sample-size and in different doses involving both cholera and non-cholera diarrhoea in children and adults are warranted to establish the efficacy of Salovum egg powder containing AFs.

## ACKNOWLEDGEMENTS

The study was conducted with the support (Grant No. GR-00339) from Novartis Consumer Health. icddr,b acknowledges with gratitude the commitment of Novartis Consumer Health to its research efforts.
